# Pediatric Brain Maturation and Migration Disorders

**DOI:** 10.3390/diagnostics12051123

**Published:** 2022-04-30

**Authors:** Matthew J. Barkovich

**Affiliations:** Department of Radiology and Biomedical Imaging, University of California, San Francisco, 505 Parnassus Ave. M-391, San Francisco, CA 94143, USA; matthew.barkovich@ucsf.edu

**Keywords:** neurodevelopmental disorders, migration disorders, brain development

## Abstract

Neurodevelopmental disorders, including neuronal migration disorders, are best understood in the context of altered normal development. Neurons normally migrate from their site of origin to their (usually cortical) destination using a wide range of molecular and cellular signaling as a guide. In the case of abnormal migration neurons: (1) do not migrate and remain at their site of origin; (2) incompletely migrate and remain within the white matter; (3) migrate to the cortex but fail to organize correctly; or (4) over-migrate, beyond the cortex. In this review, we discuss normal brain development, along with the malformations that result from these different migration abnormalities.

## 1. Introduction

The understanding of normal brain development and malformations of brain development go hand in hand, as most malformations are best understood in the context of brain maldevelopment. We discussed normal brain development from early embryogenesis to postnatal life, with an emphasis on the stages of development amenable to imaging evaluation.

## 2. Brain Development

### 2.1. Basic Embryology

During early embryogenesis, the primitive neural plate forms, displacing (and situated cranial to) the regressing primitive streak in the embryonic disk [1]. The neural plate folds at 18–23 post-conceptional days, forming the neural tube. During this process, the neural tube separates from the adjacent ectoderm, completing the process of primary neurulation [2]. The cranial and caudal neuropores become progressively smaller, and close completely at 24 and 26 post-conceptional days, respectively. The first components of the developing brain form from the closed neural tube, with the primary vesicles (the prosencephalon, mesencephalon, and rhombencephalon) arising from the cranial aspect of the closed neural tube at 4 post-conceptional weeks. At around 5 post-conceptional weeks, the secondary vesicles (the prosencephalon becoming the telencephalon and diencephalon, with the rhombencephalon becoming the metencephalon and myelencephalon) form from the primary vesicles. The caudal cell mass (the progenitor of the conus medullaris, cauda equina, and much of the sacrum and coccyx), which is initially an undifferentiated cell mass in the regressing primitive streak, begins to form the caudal neural elements during secondary neurulation [1]. This process is completed by 8 post-conceptional weeks.

### 2.2. Cortical Development and Neuronal Migration

**Figure 1:**
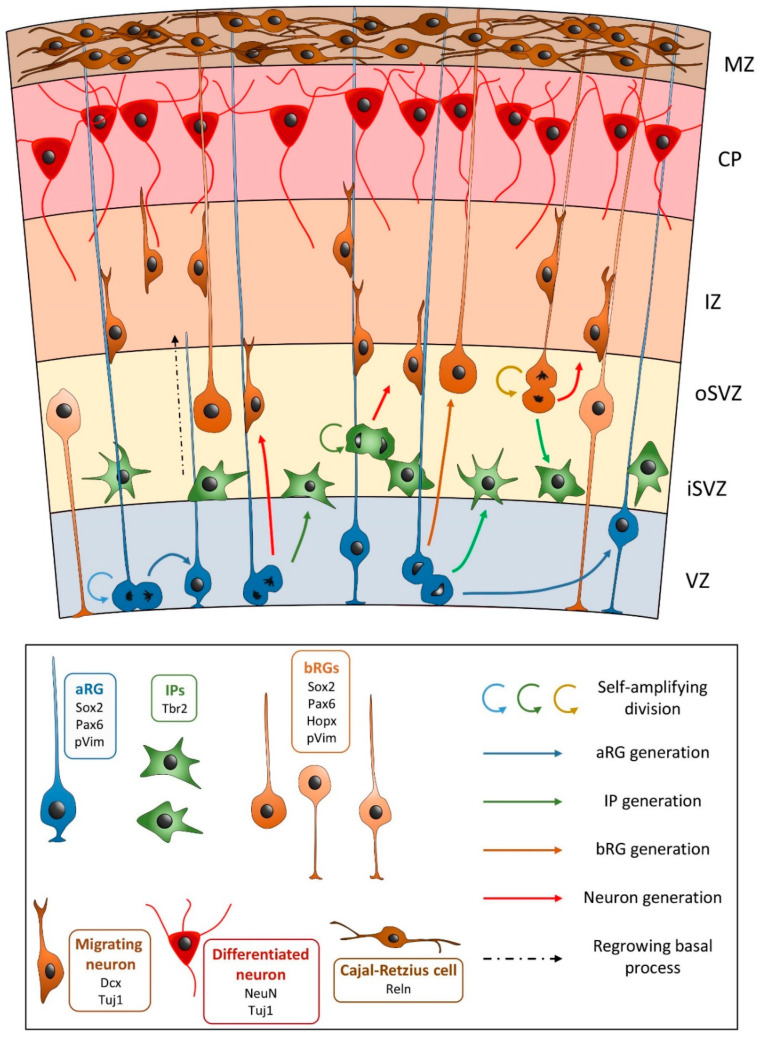
Schematic diagram of neuronal migration along apical and basal radial glial (aRG and bRG) from the ventricular zone (VZ), through the subventricular zone (SVZ), and intermediate zone (IZ) to the cortical plate (CP). The pial limiting membrane overlies the marginal zone (MZ). Outer subventricular zone (oSVZ), inner subventricular zone (iSVZ). Reproduced under a Creative Commons license from Penisson et al. Front Cell Neurosci 2019 [6].

The cerebral cortex develops within the telencephalon, which gives rise to the cerebral hemispheres. The glutamatergic, or excitatory, neurons arise from the primary germinal (ventricular) zones, and migrate directly from the ventricular zones to the developing cortex, along radial glial cells (Figure 1) [3,4]. The radial glial cells attach to the pial limiting membrane (glia limitans) that overlies the developing cortex, and which serves as the boundary/demarcation of the outer extent of the cortex [1,4]. The inhibitory (GABAergic) neurons arise from the ganglionic eminences, and migrate around the ventricles to the basal ganglia (perpendicular to the radial glia) [5]. The cerebral cortex develops “inside out”, with the earliest migrating neurons forming the deepest cortical layers. Interestingly, the radial glia may play a role in brain development that extends beyond neuronal migration, and may also be involved in axonal pathfinding and neuronal connectivity [3].

### 2.3. Commissure Formation

**Figure 2:**
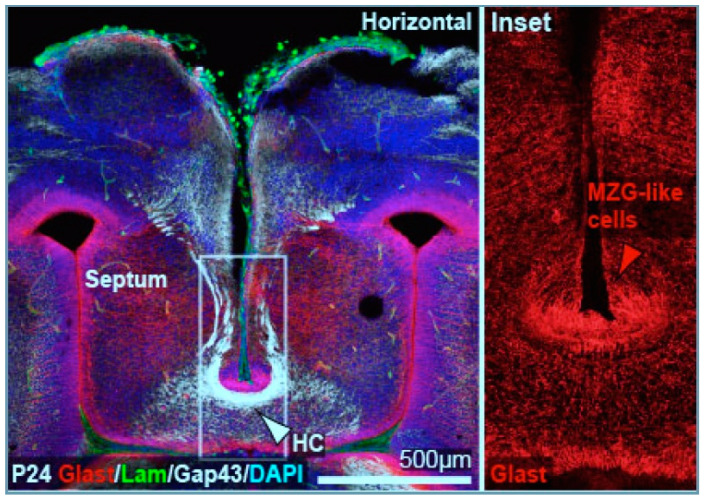
Initial callosal axons do not cross the interhemispheric fissure but instead use midline zipper glia (MZG) as a substrate to cross midline. The hippocampal commissure (HC) is seen underlying the MZG in the left panel. Reproduced under Creative Commons license from Gobius et al. Cell Reports 2016 [11].

The cerebral commissures begin developing in around the 7th gestational week, when specialized glial cells, known as midline zipper glia (MZG), migrate into the interhemispheric fissure near the junction between the telencephalon and diencephalon [4,7,8]. The MZG from each hemisphere extend multiple processes through the leptomeninges, and form a structure referred to as a “glial sling” [8,9] (Figure 2). The MZG within the glial sling secrete a variety of signaling chemicals and growth factors that guide the developing axons across the midline. Fibroblast growth factor 8 (FGF8) signaling is a major contributor to the development of the glial sling, as well as axonal pathfinding across the glial sling [10]. Alpha-tubulin is also a major contributor [7]. By 13 weeks gestational age, the major commissures are formed, with the anterior commissure and hippocampal commissure largely complete. The corpus callosum is present at 13 gestational weeks but is diminutive, and in images appears to be comprised largely of the genu and anterior body. This is not, in fact, the case, as the corpus callosum actually grows from posterior to anterior, mirroring the posterior to anterior development of the cerebral hemispheres [4]. The apparent anterior to posterior growth, seen on imaging, is likely due to the posterior displacement of the hippocampal commissure by the developing corpus callosum. At term, the splenium is often inseparable from the hippocampal commissure on midline sagittal images. Advanced MR diffusion imaging techniques such as NODDI, although not currently used in clinical practice, have provided further insight into corpus callosum development, with changes in microstructural metrics such as apparent fiber density increasing markedly in the posterior body of the corpus callosum during puberty.

### 2.4. Fetal Brain Development

Ultrasonography is the mainstay of fetal imaging and, in clinical practice, brain development is most commonly assessed using fetal ultrasonography and biometry at 20 weeks gestation. Sulcation is minimal at 20 weeks, and myelination is not well assessed by sonography, so ventricle size, head circumference, and biparietal diameter are the major biometric assessments [12,13,14]. Posterior fossa morphology, and the presence of the cavum septum, are also assessed using screening sonography. In fetuses at high risk for neurologic injury, CNS malformation, or delayed maturation, fetal MR provides important anatomic detail, as well as longitudinal assessment. Sulcation is assessed subjectively by fetal MRI, and it proceeds in an orderly and sequential fashion (Table 1), starting with the sylvian fissure (first developing around 16–20 weeks gestation), proceeding to the calcarine, parieto-occipital, and cingulate sulci (developing around 20–22 weeks), and then the central, interparietal, and superior temporal sulci (developing by 25 weeks) [4]. The hippocampal sulci often develop asymmetrically over a wider range of gestational ages (16–23 weeks) than most other sulci [4]. The general pattern of sulcation matches the pattern of brain maturation, with development occurring first in structures involved in the sensorimotor and visual pathways, and proceeding most slowly in the anterior frontal and anterior temporal regions. Sulcation can also be evaluated quantitatively [15], but, for the time being, this remains practical only in a research environment. Myelination occurs primarily on a microstructural level during fetal brain development, but is challenging to identify using fetal MRI. Myelination is discussed in the subsequent section on postnatal brain development.

### 2.5. Postnatal Brain Development

At term, all primary, secondary, and tertiary sulci are present and visible by MRI, although they have not yet developed complete adult depth and complexity. Final gyral development in the frontobasal, frontopolar, and anterior temporal regions continues into postnatal life. Additionally, prematurely born neonates have more mature gyral patterns than fetuses of a comparable gestational age [16]. Brain maturation in the postnatal period is generally identified on clinical imaging by changes in MR signal related to myelination. The developing neonatal brain progresses from appearing largely unmyelinated at term, to completely myelinated (by MR imaging) at around two years of age (Table 2). For interested readers, chapter 2 of Barkovich et al. [4] has an excellent pictural reference. The white matter of the largely unmyelinated neonatal brain has MR signal characteristics reflecting its high water content, with high signal on T2-weighted images, and low signal on T1-weighted images, relative to grey matter. The mature, fully myelinated brain has high signal in the white matter on T1-weighted images, and low signal on T2-weighted images, reflecting the displacement of water by fatty myelin. Myelination is more readily apparent on T1-weighted images during the first year of life, and more readily apparent on T2-weighted images during the second year of life. The brain has a near adult appearance on T1-weighted images at around the age of 1 year. At term, high T1 signal is seen (referred to as myelin from this point forward, for the sake of simplicity) in the posterior fossa in the medial and lateral lemniscus, medial longitudinal fasciculus, and inferior and superior cerebellar peduncles [17]. The ventral lateral thalamus, globus pallidus, and posterior portion of the posterior limbs of the internal capsules appear myelinated on T1-weighted images in term neonates. Conceptually, myelination progresses more quickly in structures within systems that are used in early life, and more slowly in structures within systems that are not used until later life. In general, myelination progresses from central to peripheral, from caudal to rostral, and from dorsal to ventral. Most sensory pathways myelinate prior to motor pathways.

After myelination is complete on T2-weighted imaging, at approximately 2 years of age, there is little change in brain morphology or signal characteristics on conventional MR sequences. Slight T2 hyperintensity within the white matter about the atria of the lateral ventricles is seen well into the second decade of life. These regions are labeled “terminal zones of myelination” [18], as axons in this area do not stain for myelin when evaluated histopathologically, even into the fourth decade of life. While this may seem to be merely an interesting piece of developmental trivia, signal abnormality related to white matter injury of prematurity occurs in a very similar location, so this normal imaging feature can be a pathological mimic. Ongoing myelination, synaptic pruning, and brain maturation can be evaluated after two years of life, using advanced MR techniques such as NODDI [19,20] (to evaluate neurite dispersion and density), or myelin-weighted imaging [15,21] (such as that derived from synthetic MRI [22]). These techniques are not currently used in clinical practice, but may be adopted for clinical use in the future. Biometry does not currently have a role in the clinical evaluation of postnatal brain maturation.

### 2.6. Pituitary Development

The pituitary gland is derived from two different embryonic tissues lines. The adenohypophysis, or anterior pituitary, arises from an extension of oral ectoderm called Rathke’s pouch, which extends superiorly and joins with an inferior extension of the neural ectoderm called the infundibulum. These two structures join below the developing cerebral hemispheres and the residual stalk of Rathke’s pouch regresses. The anterior pituitary and posterior pituitary, or neurohypophysis (arising from neural ectoderm), remain connected to the hypothalamus by the residual pituitary infundibulum.

## 3. Migration Disorders

Neurodevelopmental disorders are usually best characterized by the stage of brain development that was disrupted (when this is known or possible to determine). Migration disorders are a subset of neurodevelopmental disorders that result from abnormal neuronal migration. Neurons may:

1. Not migrate and remain at their site of origin (adjacent to the lateral ventricles);
2. Incompletely migrate and remain within the white matter;
3. Migrate to the cortex but fail to organize correctly; or
4. Over-migrate beyond the cortex.

The migration abnormality can be focal, regional, or diffuse depending on the underlying etiology (germline genetic, somatic mutation, or injury/insult), and the gene/pathway affected. Patients with somatic mutations that cause their migrational disorder can be CNS mosaics for that mutation (the mutation then only occurs in a subset of the CNS), leading to variable phenotypes for patients with the same mutation. We review the disorders resulting from the various stages of disrupted migration, including the clinical presentation, in the following section.

### 3.1. Heterotopia

**Figure 3:**
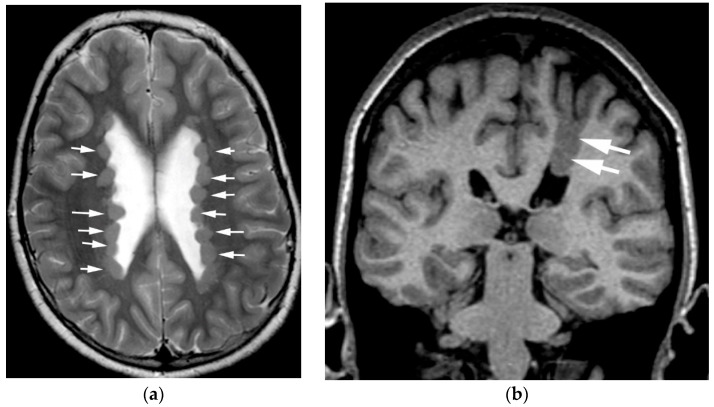
(**a**) Numerous periventricular nodular heterotopia (white arrows) are seen on this axial T2-weighted image. Note how they have the same signal intensity as cortex. (**b**) Transmantle grey matter heterotopia (white arrows) extending from the left superior frontal gyrus to the body of the left lateral ventricle seen on this coronal T1-weighted image.

With the under-migration of neurons, the cell bodies intended for the developing cortex either remain in the germinal zones (adjacent to the lateral ventricles), or are deposited in the white matter along the expected path of migration. This results from either the failure of attachment to, or premature detachment from, the radial glial cells. Periventricular nodular heterotopia (Figure 3a) appears on MRI as small nodules adjacent to the lateral ventricles that follow the signal intensity of the cortex (grey matter) on all MR sequences. Nodular heterotopia can be singular or numerous, and when seen in small numbers, or in isolation, can be an incidental finding (reported in 30% of cases, although this may not represent the true prevalence in the population) [23]. Nodular heterotopia also serve as epileptogenic foci and are one of the structural causes of medically intractable epilepsy. Band and transmantle heterotopia (Figure 3b) differ in location and extent from nodular heterotopia, but are just phenotypic variants of similar underlying pathophysiology, albeit differing in mechanism. The molecular/genetic etiologies are numerous, often related to mutations in genes for actin or tubulin, and in patients with germline genetic abnormalities, grey matter heterotopia are just one part of a multitude of other CNS malformations [16,23,24,25]. Children with isolated heterotopia are usually developmentally normal until the onset of epilepsy in the first or second decade of life.

### 3.2. Cobblestone Cortex

**Figure 4:**
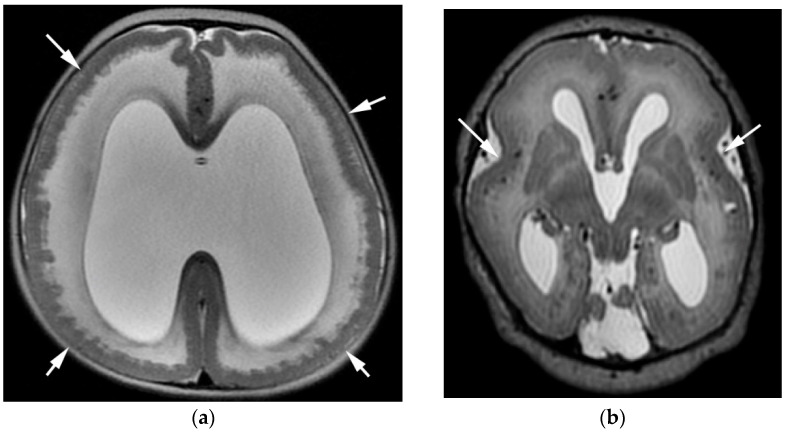
(**a**) Axial T2-weighted image of cobblestone cortex with a polymicrogyria phenotype (due to small gaps in pial limiting membrane). Note the simplified gyral pattern (white arrows). (**b**) Axial T2-weighted image of cobblestone cortex with a lissencephalic appearance, due to larger gaps in the pial limiting membrane. Note the rudimentary sylvian fissures (white arrows).

In the developing cortex, when neurons migrate successfully along the radial glial cells, they normally detach from their glial support in the appropriate cortical layer, and are demarcated from the subarachnoid space by the pial limiting membrane [26,27]. When the pial limiting membrane fails to form, or is disrupted, neurons migrate beyond the cortex through these gaps and into the subarachnoid space, leading to the thickened and disorganized cortex, called a cobblestone cortex. Despite the common cellular etiology, the macroscopic morphology varies (on imaging and gross pathologic examination) based on the size of the gaps in the pial limiting membrane [28,29]. Small gaps in the glia limitans give the appearance of multiple tiny gyri (polymicrogyria phenotype, Figure 4a), while larger gaps give an appearance of a thickened cortex (pachygyria, Figure 4b) [28]. This appearance changes as the brain matures, sulcates, and myelinates and, for that reason, these are referred to collectively as cobblestone malformations or a cobblestone cortex. The MRI appearance (polymicrogyria verses pachygyria) is of no consequence in terms of etiology or outcome, and emphasis on this descriptor, or use of the imaging phenotype as a diagnosis, is something that is best avoided. Cobblestone cortex is seen in syndromic settings, most notably in the congenital muscular dystrophies and tubulinopathies [4,28,29]. The congenital muscular dystrophies, as well as microcephalies, are their own topic and were not discussed further here. Although many patients with cobblestone cortex have genetic abnormalities as the inciting etiology, this cortical malformation can also be the result of in utero infection. TORCH infection and the Zika virus can also lead to gaps in the pial limiting membrane during neuronal migration, causing the cobblestone phenotype (usually alongside microcephaly and other malformations) [30].

### 3.3. Cortical Dysplasia

**Figure 5:**
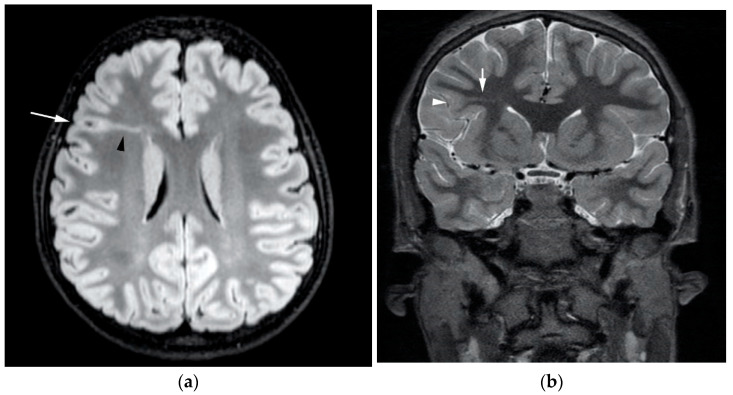
(**a**) Axial FLAIR image of a focal cortical dysplasia type IIb in the right frontal cortex with subtle blurring of grey–white differentiation (white arrow) and transmantle sign (black arrowhead), extending to the anterior body of the right lateral ventricle. The transmantle sign is diagnostic of FCD IIb. (**b**) Coronal T2-weighted image showing the same right frontal FCD IIb (white arrowhead) with transmantle sign (white arrow) seen in (**a**).

Although abnormal neurons, and a disorganized and disordered cortex, do not directly result from abnormal migration of neurons along radial glial cells, cortical dysplasia in the context of abnormal neuronal migration are still worthy of discussion in this context. Focal cortical dysplasia (FCD) are a class of developmentally unrelated cortical malformations, characterized by abnormal neuronal morphology and disorganized cortical layers. FCDs are the most common cause of pediatric medically refractory focal epilepsy [31]. FCDs are classified into several histopathologic subtypes [32], with FCD type I characterized by disturbances in either the radial or tangential (or both) arrangement of cortical neurons; FCD type II have dysmorphic neurons (and balloon cells in FCD IIb) with more profound architectural disturbances; FCD type III are best thought of as a subset of FCD I, associated with some other structural abnormality (IIIa with hippocampal sclerosis, IIIb with glial tumor, IIIc with vascular malformation, and IIId with scarring from prior injury). FCD type I are not generally identifiable using conventional MR imaging, although this may be changing with higher MR field strength and advanced imaging techniques. Advanced imaging techniques such as NODDI, although not currently in general clinical practice, have a higher sensitivity for FCDs by identifying regional differences in neurite density. FCD type II are generally the subtype identified prospectively by clinical MRI. The appearance by imaging is of blurring of the grey–white junction on both T2- and T1-weighted sequences. This is challenging to distinguish from partial volume averaging of adjacent cortex within the undulations of a gyrus, so evaluation of a suspected FCD in multiple anatomic planes with thin section, high-resolution imaging is key. Confirmatory clinical data, such as seizure semiology, localization by EEG, advanced imaging such as NODDI, or co-registration with data from magnetoencephalography are helpful in identifying these subtle cortical dysplasia. The presence of a T2 hyperintense structure extending from the depth of a sulcus to the lateral margin of the lateral ventricle is dubbed the “transmantle sign”, and is a specific imaging sign for FCD IIb [32] (Figure 5). FCD II are the most well understood in terms of inciting etiology, and appear to be sequela of mTOR/AKT/PI3K pathway dysregulation [31,33]. The histopathology of FCD II is quite similar to that of the cortical tubers in patients with tuberous sclerosis complex (TSC), another disorder related to mTOR pathway dysregulation [31,33]. FCD II are also histologically quite similar to dysplastic megalencephaly (DME), also known as hemimegalencephaly, a cerebral overgrowth disorder related to mTOR pathway dysregulation [4]. In summary, FCD II is considered a localized or focal version of the cortical abnormality seen in DME and TSC.

### 3.4. Commissural Abnormalities

The development of the cerebral commissures can be altered or disrupted in numerous ways, leading to a wide variety of abnormal commissural phenotypes. The most visually apparent phenotype, that of complete agenesis of the corpus callosum (CC), often results in a clinical phenotype that is milder than cases of CC hypogenesis, when it is an isolated malformation [34,35]. In this discussion, we briefly mentioned that commissural abnormalities are often secondary to injury to the cerebral hemispheres. In the event of cerebral injury, there are no resultant white matter tracts from the area of injury that need to cross the midline. Commissural abnormalities are associated with midline cysts or lipomas; these structures can be quite large, exerting mass effect on adjacent structures [36]. Callosal abnormalities are associated with many syndromes and, in the case of syndromic commissural abnormalities, the clinical features of the syndrome define the clinical phenotype to a far greater degree than the morphology of the commissural abnormality [35,37]. These cases of secondary CC hypogenesis have little relation to altered axonal migration or pathfinding, and are not discussed further.

### 3.5. Agenesis of the Corpus Callosum

**Figure 6:**
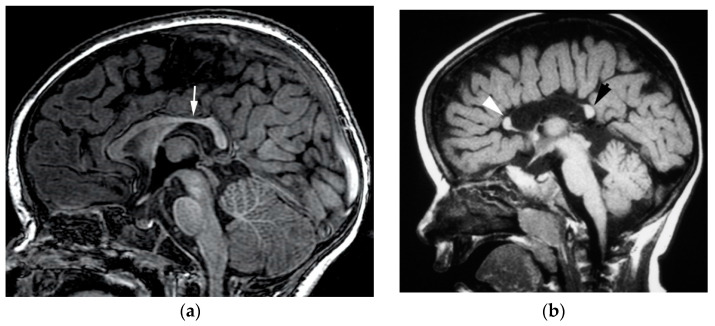
(**a**): Normal midline sagittal T1-weighted image of fully developed corpus callosum. Note the slight thinning of the posterior body (white arrow), which is normal. (**b**): Midline sagittal T1-weighted image of corpus callosum agenesis. The two midline commissures seen anteriorly and posteriorly are the anterior commissure (white arrowhead) and hippocampal commissure (black arrow) respectively. These would not be present in tricommissural agenesis.

In complete or tricommissural agenesis, neither the hippocampal nor anterior commissure develop, in addition to the corpus callosum being absent. Tricommissural agenesis is rare, and the clinical phenotype and overall severity of disability is more strongly correlated with the associated brain malformations (present in 50% of patients with CC agenesis) rather than the commissural abnormality itself [4,37]. In the absence of the CC, the leaves of the septum pellucidum extend to the medial edge of the limbic lobe, and callosal axons run with the pellucidum, forming Probst bundles (best seen on coronal images). When there is complete agenesis of the CC, but the hippocampal commissure and anterior commissure are present (isolated callosal agenesis) (Figure 6), the hippocampal commissure is enlarged and mimics a section of the CC on midline sagittal images. Without a normally developed CC, the cingulate gyri do not form normally and, thus, the cingulate sulci do not form. The bodies of the lateral ventricles have a straight and parallel configuration, due to the absence of CC. The morphologic differences in the lateral ventricles and cingulate gyri are simply secondary to the absent structure, and are not of any functional consequence [4,37].

### 3.6. Hypogenesis of the Corpus Callosum

Hypogenesis of the corpus callosum is most frequently the result of a developmental injury of the cerebral hemispheres, or another inciting cortical malformation. The severity of clinical phenotypes in patients with isolated CC hypogenesis can paradoxically be more severe than those with complete agenesis [34,35,37]. In disorders such as lobar or middle interhemispheric variant holoprosencephaly, where there is a failure of midline differentiation [38], the glial sling fails to develop in regions where midline does not differentiate, and therefore, the CC does not form in those areas. The hypogenesis of CC is not associated with Probst bundles or abnormal ventricular morphology. Normal thinning of the posterior body (isthmus) of the corpus callosum should not be mistaken for CC hypogenesis (Figure 6a).

## 4. Conclusions

Understanding normal brain development is critical for the interpretation of normal imaging findings, and also offers insight into brain function. In addition, neurodevelopmental disorders are often best understood in the context of the developmental processes that are disrupted. In this review of disorders of neuronal migration, we emphasized the critical process of in utero cortical development, specifically the migration of neurons along radial glial cells from the germinal zones to the developing cortex. Most migration disorders are attributed to perturbations of this critical process, leading to either neuronal under-migration (and resultant grey matter heterotopia), or over-migration (and disorders such as cobblestone cortex).

## Figures and Tables

**Table 1:** Sulcation of the brain.

Sulcus	Gestation Age Seen on Fetal MRI
Sylvian	16 wk
Hippocampal	16–23 wk
Calcarine	20–22 wk
Parieto-occipital	20–22 wk
Cingulate	20–22 wk
Collateral	23–26 wk
Rolandic	25 wk
Interparietal	25 wk
Superior temporal	25 wk
Precentral	24–28 wk
Postcentral	24–28 wk
Superior frontal	24–28 wk
Middle temporal	24–28 wk
Primary fissure of cerebellum	25–28 wk

**Table 2:** Ages When Changes of Myelination Appear.

Anatomic Region	T1-Weighted Images	T2-Weighted Images
Superior cerebellar peduncle	28 gest wk	37 gest wk
Median longitudinal fasciculus	25 gest wk	29 gest wk
Medial lemnisci	27 gest wk	30 gest wk
Lateral lemnisci	26 gest wk	27 gest wk
Middle cerebellar peduncle	Birth	Birth to 2 mo
Cerebral white matter	Birth to 4 mo	3–5 mo
Posterior limb internal capsule Anterior portion Posterior portion	First month 36 gest wk	4–7 mo 40 gest wk
Anterior limb internal capsule	2–3 mo	7–11 mo
Genu corpus callosum	4–6 mo	5–8 mo
Splenium corpus callosum	3–4 mo	4–6 mo
Occipital white matter Deep Subcortical	3–5 mo 4–7 mo	9–14 mo 11–15 mo
Midfrontal white matter Deep Subcortical	3–6 mo 7–11 mo	11–16 mo 14–18 mo
Anterior frontal white matter Deep Subcortical	5–8 mo 10–15 mo	12–18 mo 24–30 mo
Centrum semiovale	2–4 mo	7–11 mo

Reproduced with permission of the publisher [4].

## Data Availability

Not applicable.
